# Agar lot-specific inhibition in the plating efficiency of yeast spores and cells

**DOI:** 10.1093/g3journal/jkae229

**Published:** 2024-09-23

**Authors:** Reine U Protacio, Mari K Davidson, Emory G Malone, Dominique Helmlinger, Jeremy R Smith, Patrick A Gibney, Wayne P Wahls

**Affiliations:** Department of Biochemistry and Molecular Biology, University of Arkansas for Medical Sciences, Little Rock, AR 72205-7199, USA; Department of Biochemistry and Molecular Biology, University of Arkansas for Medical Sciences, Little Rock, AR 72205-7199, USA; Department of Biochemistry and Molecular Biology, University of Arkansas for Medical Sciences, Little Rock, AR 72205-7199, USA; Centre de Recherche en Biologie Cellulaire de Montpellier, CNRS, University of Montpellier, 34293 Montpellier Cedex 05, France; Department of Food Science, Cornell University, Ithaca, NY 14853-7201, USA; Department of Food Science, Cornell University, Ithaca, NY 14853-7201, USA; Department of Biochemistry and Molecular Biology, University of Arkansas for Medical Sciences, Little Rock, AR 72205-7199, USA

**Keywords:** *Schizosaccharomyces pombe*, *Saccharomyces cerevisiae*, agar, efficiency of plating, toxicity, quality control

## Abstract

The fission yeast *Schizosaccharomyces pombe* and the budding yeast *Saccharomyces cerevisiae* are highly diverged (530 mya), single-celled, and model eukaryotic organisms. Scientists employ mating, meiosis, and the plating of ascospores and cells to generate strains with novel genotypes and to discover biological processes. Our 3 laboratories encountered independently sudden-onset, major impediments to such research. Spore suspensions and vegetative cells no longer plated effectively on minimal media. By systematically analyzing multiple different media components from multiple different suppliers, we identified the source of the problem. Specific lots of agar were toxic. We report that this sporadic toxicity affects independently the agar stocks of multiple vendors, has occurred repeatedly over at least 3 decades, and extends to species in highly diverged taxa. Interestingly, the inhibitory effects displayed variable penetrance and were attenuated on rich media. Consequently, quality control checks that use only rich media can provide false assurances on the quality of the agar. Lastly, we describe likely sources of the toxicity and we provide specific guidance for quality control measures that should be applied by all vendors as preconditions for their sale of agar.

## Graphical Abstract

**Graphical Abstract:**
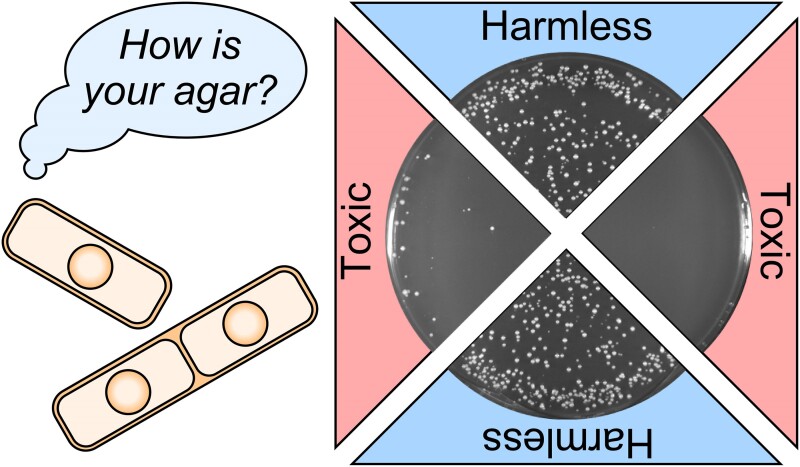

## Introduction

Agar is a hydrocolloidial gelling agent that is extracted from the cell walls of red algae (phylum *Rhodopyta*), often from numerous different species in the genera *Gracilaria* and *Gelidium* (which together have more than 500 recognized species) (Porse and Rudolph 2017; Vandepitte *et al*. 2018; Borg *et al*. 2023). Agar is prepared by a crude extraction process that includes hot water extraction, gelling, drying, and milling; refinements often include alkaline hydrolysis to improve yield and quality. Agar is composed primarily of agarose (heterogeneous polymers of the disaccharide agarobiose) and agaropectin (a heterogeneous mixture of galactan polymers). It is a particularly useful gelling agent because it displays hysteresis, with a gelling temperature around 40°C and a melting temperature around 85°C.

The primary use of agar is in the food industry, where it is employed to stabilize, thicken, gel, or otherwise modify the textural properties of food items; it also provides dietary fiber (Bose *et al*. 2023). The second most common use for agar, which consumes about 10% of the world's supply (Kim *et al*. 2017; Porse and Rudolph 2017), is to solidify culture media for the isolation, cultivation, and analyses of microorganisms (Koch 1882). Additional biological and biomedical applications include cell motility assays (Partridge and Harshey 2013); plant propagation (Ma *et al*. 2023), drug screening (Bidaud *et al*. 2021); and analyzing the invasive potential of cancer cells (Wang 2023). Agar can also be used in hydrogel films (Ma *et al*. 2023); for applications such as wound dressings (Shen *et al*. 2021); for environmental remediation like adsorption of heavy metals from waste water (Rani *et al*. 2018); as nanoparticles for drug delivery (Gaikwad *et al*. 2024); and as scaffolds for immunoassays (Hornbeck 2017).

Hundreds of research laboratories use agar plates to propagate and study outstanding model eukaryotic organisms, such as the fission yeast *Schizosaccharomyces pombe* (Harris *et al*. 2022) and the budding yeast *Saccharomyces cerevisiae* (Wong *et al*. 2023). Although they are both called “yeast,” these 2 single-celled eukaryotes latest common ancestor was about 530 mya (Shen *et al*. 2020), which allows one to compare the conservation and divergence of fundamental biological processes across taxa (Wood *et al*. 2018). Interestingly, many properties of fission yeast biology more closely resemble those of humans (Vyas *et al*. 2021), perhaps due to accelerated evolution in the budding yeast lineage (Shen *et al*. 2020). Fission yeast can be cultured as a haploid or as a diploid and these 2 states can be easily interconverted by mating and meiosis (Ohtsuka *et al*. 2022; Kawamukai 2024). This supports powerful genetic approaches that are complemented by equally powerful molecular tools [e.g. (Gao *et al*. 2014; Storey *et al*. 2019; Torres-Garcia *et al*. 2020; Billmyre *et al*. 2022; Ishikawa and Saitoh 2023)], which has made fission yeast an eminent model to study a variety of broadly conserved eukaryotic processes [see (Hoffman *et al*. 2015; Nurse 2020; Harris *et al*. 2022; Rutherford *et al*. 2024) and refs therein]. For example, we use this model organism to discover mechanisms by which specific DNA sequences, their binding proteins, and chromatin remodeling factors control the positioning of meiotic recombination throughout the genome (Storey *et al*. 2018; Mukiza *et al*. 2019; Protacio *et al*. 2022). In our line of research—as in many other areas of research using diverse organisms—we rely heavily on being able to measure with precision the titers of viable cells.

Here, we report sudden-onset reductions in the plating efficiency of fission yeast spores and cells which had catastrophic impacts on the progress of our research programs. We describe how we traced that problem to sporadic toxicity within specific batches of agar from different suppliers. We show that agar lot-specific inhibition of growth applies to other model species, such as budding yeast. We discuss likely sources for this toxicity and we provide specific guidance for quality control measures that should be applied by vendors as preconditions for their sale of agar.

## Materials and methods

### Yeast strains and genotypes

The names and genotypes of fission yeast *S. pombe* strains used in this study are: WSP 3776 (*h^−^*), WSP 5819 (*h^+^ ade6-M375*), WSP 7850 (*h^−^ ade6-3049*), and DHP 148 (*h^90^*). Spore suspensions were from crosses between WSP 5819 and WSP 7850. For budding yeast *S. cerevisiae*, we used a GAL^+^ prototrophic diploid derivative of a standard laboratory strain lineage, S288C, in which the common transposon insertion in the *HAP1* gene was repaired. This wild-type laboratory strain, DBY12007 (*HAP1*^+^/*HAP1*^+^ prototrophic diploid), was originally constructed in Fred Winston's Lab where it is named FY2648 (Hickman and Winston 2007; Hickman *et al*. 2011).

### Culture media and methods

Standard formulations were used for fission yeast synthetic minimal media (Edinburg minimal media, EMM), minimal media (nitrogen base, NB), rich media (YE), and sporulation media (SP) (Gutz *et al*. 1974; Wahls *et al*. 1993; Kon *et al*. 1998; Forsburg and Rhind 2006). An “L” or “A” is included in the name to designate liquid or agar (solid) media, respectively (e.g. NBA is nitrogen-base agar). When required to support the growth of adenine-auxotrophic strains, adenine was included in the minimal media at 100 µg/ml (Gutz 1971). Budding yeast cells were cultured using standard rich and minimal media formulations, including YPD and synthetic defined (SD) (Sherman 2002). Solid media formulations included 2% w/v agar. Standard methods were used for yeast culture, for genetic crosses to induce mating and meiosis, and for the preparation of ascospore suspensions (Kon *et al*. 1998; Gao *et al*. 2008). Numbers of cells or spores in suspensions were determined using a hemocytometer. Culture plates for fission yeast were incubated at 32°C for 3–4 days; those for budding yeast were incubated at 30°C for 2–3 days.

### Statistical measures

Efficiencies of plating (EOP, expressed as viable fraction or percent viable) are based on number of observed colonies divided by number of cells or spores plated. The test between 2 proportions was used to calculate *P-*value of differences in EOP between pairs of plates; two-sided *t*-test was used for differences in EOP between series of plates. Compared EOP values which had *P* ≤ 0.05 were judged to be significantly different from each other.

## Results

In this study, we used 3 types of culture media that are broadly employed by the fission yeast research community. Our rich media was yeast extract liquid (YEL) or agar (YEA); for minimal media we used nitrogen-base liquid (NBL) or agar (NBA), as well as Edinburgh minimal media liquid (EMML) or agar (EMMA). The only difference between the liquid and solid media of each type was the inclusion of 2% agar in the latter. We also used a similar formulation of a NB media for budding yeast (SD and SDA). We describe below convergent discoveries made independently by 3 separate laboratories, 2 using fission yeast and 1 using budding yeast.

### Nomenclature in the paper

The word “lot” is used to designate a specific, individual media component (e.g. agar) whose source is traceably distinct from that of other lots. The distinct lots are uniquely identified within the paper by the name of the supplier and, to the extent possible, by supplier-designated lot numbers. With regard to culture media, the word “batch” denotes a single, homogeneous preparation of media, including the collection of Petri plates that were cast using that single preparation. Separate batches of media can have identical formulations and identical lots for each component (e.g. to test for reproducibility); or identical formulations but different lots for a given component (e.g. to isolate supplier-specific or lot-dependent effects); or different formulations (e.g. to compare impacts of minimal and rich media).

### Sudden-onset reduction in the plating efficiency of spores

**Fig. 1.**
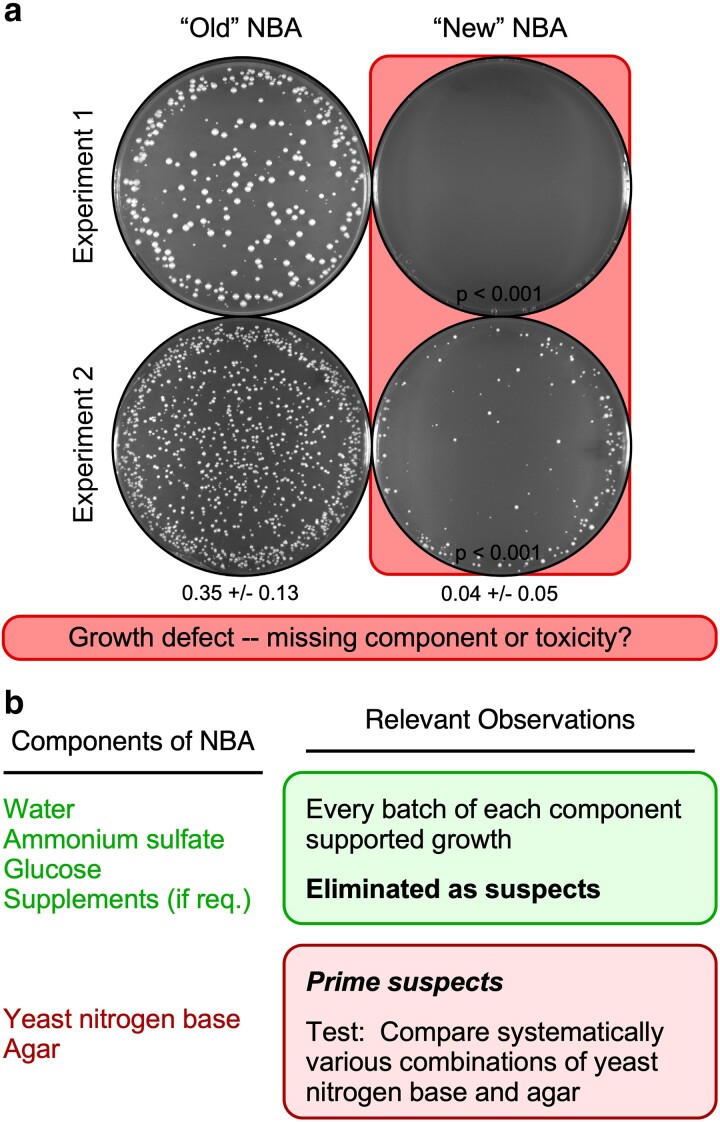
Fission yeast spores plate less efficiently on some batches of NBA media than on others. a) Images show representative results obtained with 4 different batches (i.e. separate preparations) of NBA that were prepared contemporaneously. The batch labels (*old* and *new*) reflect the relative order in which the media components were purchased. Within each experiment, aliquots of the identical spore suspension (from mating of strains WSP 5189 and WSP 7850) were plated in parallel on 2 different batches of media (1,290 spores per plate in experiment 1 and 2,500 per plate in experiment 2). Background highlights (*coral* color) emphasize the lower EOP on one batch of media vs the other. Here and in subsequent figures, inset *P*-values were calculated from EOP value vs that in column 1; column statistics values are mean and SD. b) Rationale and approach. Diagram lists components of NBA media; empirical analyses of individual components focused attention on the ones most likely to be responsible for the media batch-specific differences in EOP.

In July of 2023, we encountered a major, puzzling impediment to our research using fission yeast. Plating a known number of spores on NBA media yielded extremely low colony titers (e.g. Fig. 1a). This low EOP was severe, widespread, and reproducible; it affected multiple different batches of media that were prepared by 3 different scientists. The problems were chronic and persisted over a period of nearly 3 months before the cause of the problem was isolated (as described below). The low EOP affected equally experiments that involved spores with numerous different genotypes (e.g. with alleles that were wild-type or mutant for a variety of *cis-*acting DNA sites and *trans*-acting factors). The chronic, widespread, reproducibly low EOP suggested that a substantial fraction of the spores were inviable or that there was a problem with the media. Since we often keep stocks of spore suspensions from previous experiments, we had available stocks with known, high titers. When we plated those viable spores on the newer NBA media they plated inefficiently, suggesting that the problem was with the media, rather than the spores.

To test whether there was a problem with the culture media, we plated spore suspensions in parallel on different batches of NBA that had identical formulations but contained media components purchased at different times or from different suppliers. The results were striking. Spores plated efficiently on our “old” NBA media, which contained components that we purchased in the past, whereas spores plated poorly on our “new” NBA media, which was made using more recently purchased components (Fig. 1a). Since the major differences in EOP occurred even when the biological samples were identical (e.g. Fig. 1a), we conclude that the differences are attributable to the media itself. Presumably, some ingredient or component in the NBA media was missing or toxic. We describe in a subsequent section how we narrowed down the list of suspects. However, for the sake of clarity, we first describe (out of chronological order) the systematic approach required to solve the mystery.

### Power and precision of the approach

**Fig. 2.**
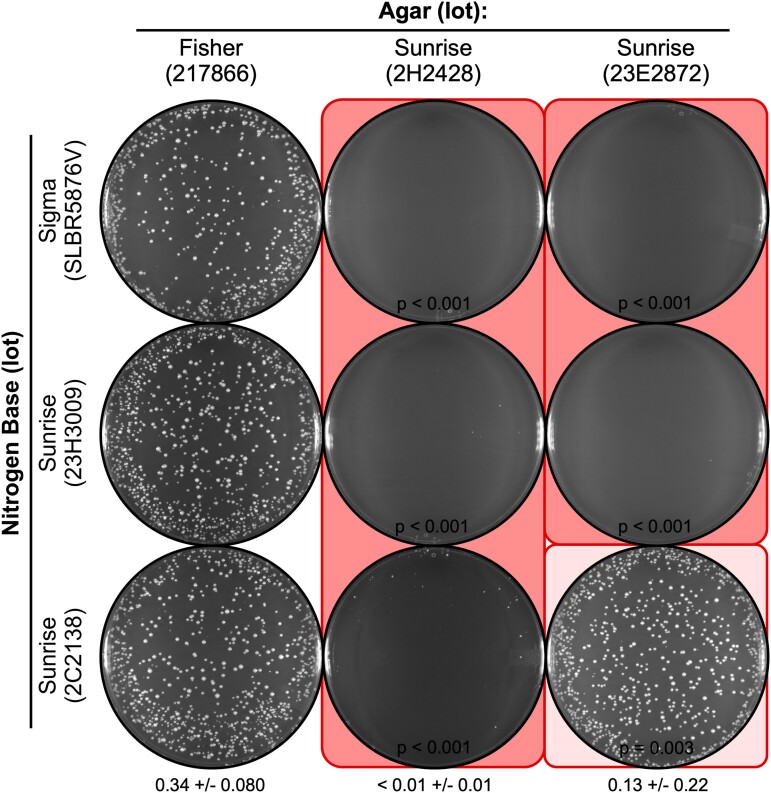
Agar lot-specific inhibition of spore colony growth on NBA. Nine different batches of NBA media were prepared using the indicated lots of NB and agar as variables. Images show EOP for representative platings of the same spore suspension (2,500 spores per plate, from WSP 5189 × WSP 7850) on the different media. Note that each lot of NB can support growth (column 1) and that a subset of the lots of agar (columns 2 and 3) reduce the EOP. In this and subsequent figures, the degree of shading (*coral* color) reflects the magnitude of the inhibitory effect.

Figure 1 exemplifies details about the approach, nature of results, and interpretations of data that apply to all of the other figures in this paper. Each figure depicts the results of an empirically based, specifically designed, well-controlled experiment in which aliquots of the *same* spore suspension (or suspension of vegetative cells) were plated in parallel on different batches of media. For example, Fig. 1a contains data from 4 different batches of media; to conduct experiments for Fig. 2, we made 9 distinct batches of media; and so on. For each batch of media in each figure panel, a representative plate image is shown; additional plates of each batch reproducibly yielded similar results. Since the same kind and number of spores (or cells) were plated, substantive differences in EOP between the different batches of media are obvious and unambiguous in the plate images, as well as in quantitative tabulations of the same data. Moreover, this approach supported the inclusion of well-matched controls: Every experiment whose data are presented in this paper contained one or more batches of media that supported efficient plating (e.g. Fig. 1a, column 1) and one or more batches of media that had compromised EOP (e.g. Fig. 1a, column 2). This allows one to calculate whether a given reduction in EOP is statistically significant. For example, in Fig. 1a the 2 “new” batches of NBA each had significantly lower EOP than the 2 “old” batches of NBA (at *P* ≤ 0.05), even though there were differences in EOP between the 2 “new” batches. The basis for such differences was elucidated as follows.

### Narrowing down the list of suspects

We prepare our NBA minimal media (and other types of culture media) from its individual components (Fig. 1b). We used 2 approaches to test whether a given component of the media was insufficient or toxic. First, if a specific batch of a given component was still available (e.g. bottle of ammonium sulfate solution), we retested that specific batch in combination with different batches of other components. Second, for a given component, we tested whether different lots or batches of that component (e.g. different bottles of ammonium sulfate) supported growth when used to make media in which the other components were maintained constant. These experiments revealed no problems with the water, ammonium sulfate, glucose, and supplements used to make the media. Every lot and every preparation of each component supported the efficient plating of spores and vegetative cells on solid media and the growth of cells in liquid cultures. We then applied a similar approach to test the prime suspects, yeast NB and agar.

### Low efficiency of spore plating is caused by the agar, not the nutrients

Since agar is a relatively inert gelling component of NBA media and the nutrients are provided by the NB (Fig. 1b), it seemed likely that the differences in EOP were due to the batch or lot of NB. To test this hypothesis, we conducted a series of experiments in which we combined systematically different lots of NB and agar, changing only one variable at a time. Moreover, because we had historically purchased much of our NB and agar from one supplier, we compared in parallel multiple different lots from that vendor, as well as lots purchased from different vendors. Interestingly, each lot of NB from each supplier supported highly efficient plating of spores (e.g. Fig. 2, left column). We conclude that each lot of NB contains all of the nutrients (other than carbon source) that are necessary and sufficient to sustain efficient spore germination and colony growth. We also conclude that those batches of NB contain no detectable inhibitors of growth. We, therefore, reject our hypothesis that the low EOP was caused by a defect or insufficiency in the NB (nutrients).

Notably, when the various lots of NB that were known to support growth were brought together with some lots of agar, the EOP fell dramatically (Fig. 2, middle and right columns). Interestingly, other lots of agar (e.g. Fig. 2, left column), including prior lots that we had purchased from our historically preferred vendor (e.g. Fig. 1a, left column), did not inhibit the EOP. We conclude that there is an agar lot-specific inhibition in the EOP of fission yeast spores on the NBA minimal media. Since agar is a solidifying agent that provides no nutrients to the culture media, we can conclude that the inhibition is due to the presence of toxic agent(s) within the affected lots. Furthermore, because this toxicity was manifest for some but not all lots of agar from the same vendor (Figs. 1 and 2), we conclude that it is a sporadic problem.

### Nutrients in the media can affect the toxicity of the agar

The experiments in which we systematically changed one variable at a time also revealed that the agar lot-specific toxicity displays variable penetrance. For example, agar from lot 23E2872 strongly suppressed the EOP of spores on NBA that contained NB from lots SLBR5876V and 23H3009 (Fig. 2, right column). However, when the same agar was in combination with NB from lot 2C2138, spores were plated efficiently (Fig. 2, right column, bottom plate). A similar result was observed for agar from lot 2H2428: When in combination with NB from lots SLBR5876V and 23H3009, this agar strongly suppress the EOP; whereas the EOP was improved slightly when this agar was in media that contained NB from lot 2C2138 (Fig. 2, middle column, bottom plate). Thus, some batches of NB can suppress the toxicity of the agar. Moreover, the same batch of NB can suppress the toxicity of multiple, different batches of agar. Interestingly, the degree of suppression varied in an agar lot-specific fashion (Fig. 2, compare bottom plates, middle, and right columns). This provides compelling evidence that some lots of agar are more toxic than other lots, which is concordant with our conclusion that some lots of agar are toxic and others are not. A similar logic applies for the suppression of toxicity by only some batches of NB (Fig. 2, row 2 vs row 3), which is likely mediated by lot-to-lot differences in the abundance of the suppressive component(s) within the NB. The richness of the nutrients in the lot of NB is a good candidate for suppression of toxicity (evidence described below).

We can summarize the preceding results and conclusions using a “poison and antidote” analogy. The effective amount of toxicity (“poison”) in the agar can vary from lot-to-lot; similarly, the amount of counteracting factor (“antidote”) in the NB can vary. Correspondingly, the EOP in the presence of the poison will be influenced by the relative amounts of both the poison and the antidote.

### A delicate balance between low EOP and high EOP

Several observations provide further insight into the nature of the variable penetrance of toxicity. First, for toxic agar lot 23E2872, different lots of NB from the same vendor differentially suppressed the toxicity (Fig. 2, right column): NB lot 23H3009 had a plating efficiency near zero, whereas lot 2C2138 supported nearly wild-type EOP. The fine balance can also be seen between lots of toxic agar (Fig. 2, bottom row). When NB lot 2C2138 was together with agar lot 2H2428, the EOP was very low; when the same NB was in combination with agar lot 23E2872, the EOP was much higher. We conclude that the variable penetrance is dictated in part by the proportionate amounts of the inferred toxin (agar lot) and the inferred counteracting agent (NB lot). However, the following observations indicate that the variable penetrance is not dictated exclusively by the proportionate amounts of the toxin and antidote.

**Fig. 3.**
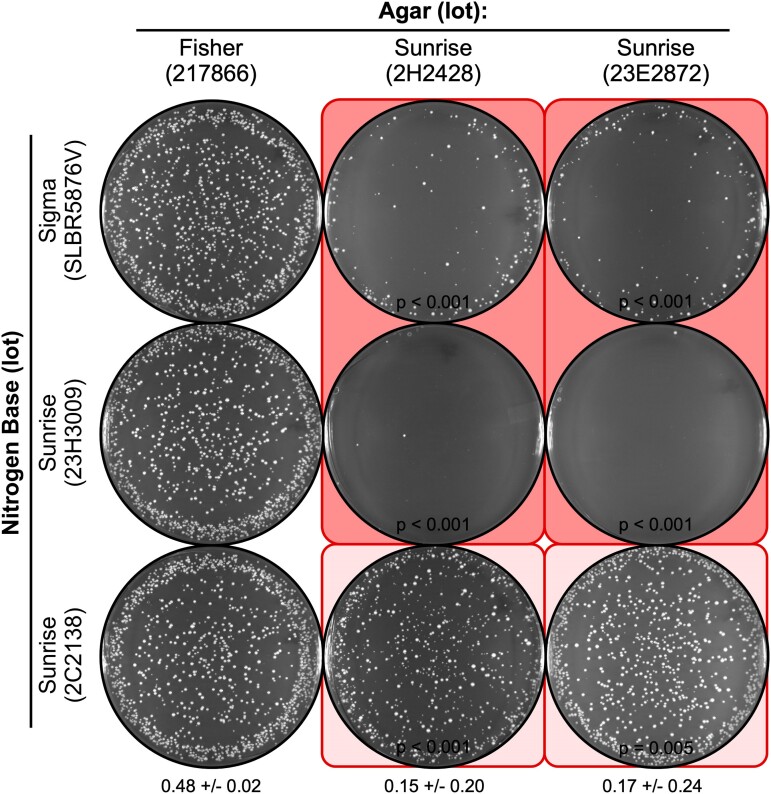
Reproducibility and variable penetrance batch-to-batch on NBA. The approach and media formulations were identical to those in Fig. 2 but employed separately prepared batches of each media. Note that the same lots of agar reproducibly reduce the EOP (columns 2 and 3). Also note that the magnitude of the inhibitory effect can vary from batch to batch; for example, the reduction in EOP for row 1, column 2 is less severe than the reduction in EOP for the same configuration in Fig. 2.

The statistically significant, agar lot-specific reduction in the plating efficiency of spores was observed reproducibly between experiments where the media formulations and individual components were identical but were prepared in separate batches (e.g. see *P-*values in Figs. 2 and 3). However, there were also significant differences in EOP within paired batches for some combinations of agar lot and NB lot. For example, when inhibitory agar from lot 2H2428 was with NB lot SLBR5876V, the EOP was either near 0 (Fig. 2, top row, column 2) or about 15% vs control (Fig. 3, top row, column 2)—even though the formulations and individual components of the 2 batches of media were identical. The same effect occurred when inhibitory agar from lot 23E2872 was with NB lot SLBR5876V; again, the control-normalized EOP was either near 0 (Fig. 2, top row, last column) or about 15% (Fig. 3, top row, last column). Similarly, for paired batches when the toxic agar lot 2H2428 was with NB lot 2C2138, the EOP was about 3% (Fig. 2, bottom row, column 2) or 78% vs control (Fig. 3, bottom row, column 2). Thus, within each of these 3 batch-to-batch comparisons, each batch conferred a statistically significant reduction in the EOP vs controls (i.e. reproducibly exhibited toxicity) but there were major differences in the magnitude of the toxicity between paired batches (> 15-fold, > 15-fold, and 26-fold differences in EOP, respectively).

We conclude that there is a delicate balance between the amounts of the inferred toxic agent in the agar and the inferred counteracting agent in the NB; moreover, this balance can apparently be tipped by additional, yet-identified factors. Be that as it may, there seems to be a fairly narrow range within which there is a dramatic change between low and high EOPs. Stated metaphorically, the identically formulated minimal media can have the appearance of either Dr Jekyll or Mr Hyde. This is particularly insidious because even minor perturbations—such as differences that might occur during the sterilization of 2 identically formulated batches of the same media—can demonstrably affect the plating efficiency (compare results in Figs. 2 vs 3). And as illustrated nicely by results described thus far, this type of variability can confound efforts to diagnose the source of problems with culture media.

### Toxicity and variable penetrance also affect the plating of vegetative cells

**Fig. 4.**
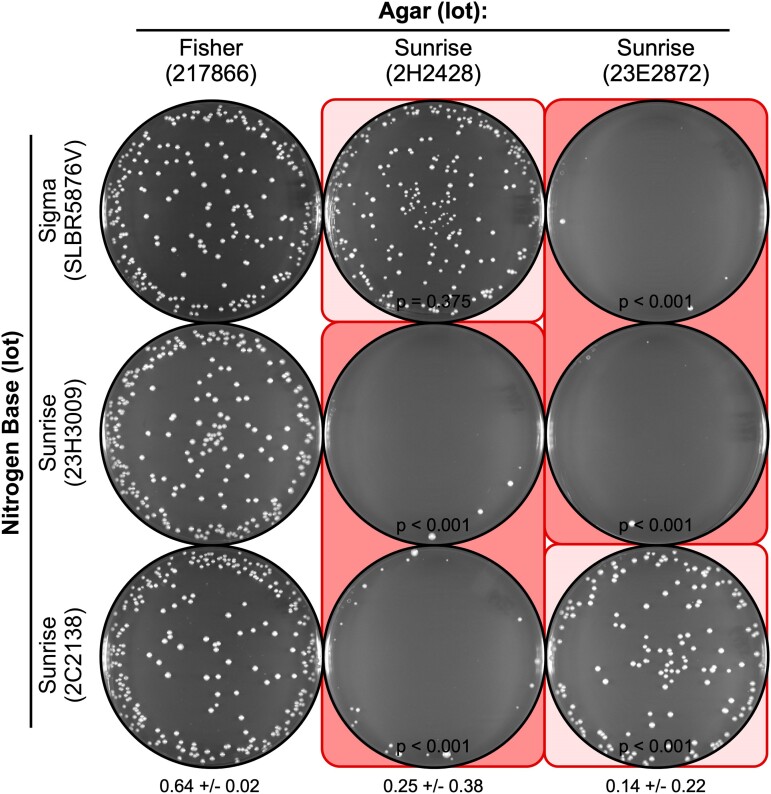
Agar lot-specific inhibition in plating of vegetative cells on NBA. Cells of strain WSP 3776 from log-phase growth in liquid culture were plated (using 400 cells per plate) on the indicated media. Note that each lot of NB can support growth (column 1) and that a subset of the lots of agar (columns 2 and 3) reduces the EOP. The patterns of inhibition for vegetative cells are like those observed for the plating of spores (Figs. 2 and 3).

The low EOP of spores on NBA media that contains toxic agar might be due to defects in spore germination or in subsequent growth of the vegetative cells. To distinguish between these 2 modes of action, we plated aliquots of cells from log-phase liquid cultures on the identical media that were used to measure the EOP of spores. The results were essentially identical: Each lot of NB from each supplier supported highly efficient plating of vegetative cells (Fig. 4, left column); one lot of agar supported high EOP (Fig. 4, left column); 2 lots of agar were toxic (Fig. 4, right 2 columns); and some lots of NB differentially suppressed the toxicity of the agar (Fig. 4, light and dark background shading). We conclude that the agar lot-specific toxicity affects vegetative cell growth, not just the plating efficiency of spores. Similarly, we conclude that the principles and mechanisms for variable penetrance (proportionate ratios of “toxin” and “antidote”) apply to both spores and vegetative cells.

### Sporadic toxicity affects lots of agar from multiple different vendors across multiple decades

**Fig. 5.**
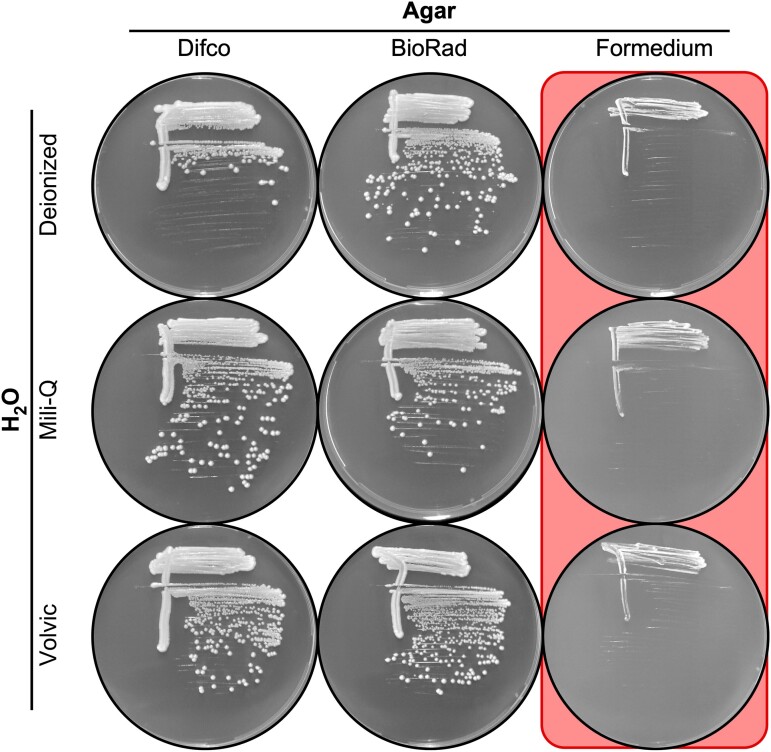
The toxicity affects agar from multiple suppliers. Nine different batches of EMMA media were prepared using the indicated lots of water and agar as the variables. Images show plating of the same strain (DHP 148) streaked out on the different media. Note that each of the nutrient components and type of H_2_O supports growth (columns 1 and 2) and that one lot of agar inhibits growth (column 3).

During a different research project in a separate laboratory, we also encountered unexpected, sudden-onset reductions in the plating efficiency of fission yeast cells, this time on EMMA. As with the process described above, we systematically tested various components of that media for potential insufficiencies or toxicities. For example, we tested, one variable at a time, all the combinations of 3 different types of water and 3 types of agar (Fig. 5). Each type of water supported growth (Fig. 5, 2 left columns), demonstrating that neither the water nor the nutrient components harbored any toxins; 2 types of agar supported growth (Fig. 5, 2 left columns); and 1 type of agar strongly suppressed the EOP (Fig. 5, right column). We conclude that toxicity occurs sporadically in a subset of agar lots from multiple different suppliers. Notably, we discovered the toxicity not only in agar lots from different vendors but also in lots whose purchase was widely separated in time (e.g. 2012 for data in Fig. 5 and 2023 for Fig. 4). This time frame provides compelling evidence that those separate instances of toxicity were of independent origin.

Our searches of scientific publication databases failed to identify papers related to adverse impacts of agar on the culture of fission yeast. Therefore, to share an advisory and to seek feedback on the potential extent of the problem, we sent a query to members of the fission yeast research community via the PombeList email server. We also posted a preprint version of this article (Davidson *et al*. 2024) and awareness was further promoted by a news article in *Science* (Herring 2024). Multiple respondents reported encountering identical, similar, or related problems with agar, affecting fission yeast and budding yeasts such as *S. cerevisiae* (e.g. Natalie Saini, personal communication) and *Candida albicans* (e.g. John McCusker, personal communication). Interestingly, the reported issues occurred in each of 3 consecutive decades and involved agar from multiple different vendors. For example, in 2006 there was apparently a batch of agar toxic to *S. pombe* that went from one wholesale source to contaminate the stocks of multiple different vendors (Nick Rhind, personal communication). Sunrise Science Products, whose agar is discussed in this article, was reportedly instrumental in identifying the toxic batch and supplying a nontoxic alternative.

### Sporadic toxicity of agar affects highly diverged species

**Fig. 6.**
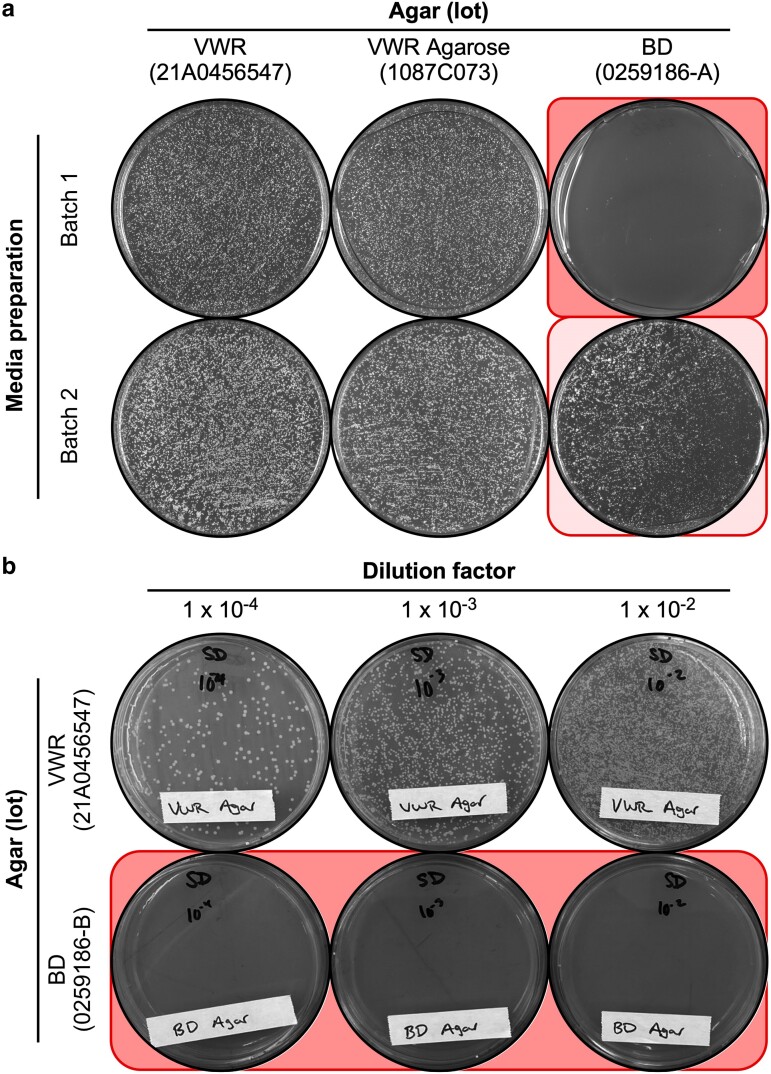
Agar lot-specific inhibition in the plating efficiency of budding yeast cells. Different batches of media were prepared as indicated and equal aliquots of cells (strain DBY12007) were plated in parallel on each batch. a) Agar lot-specific toxicity with variable penetrance. Media batches 1 and 2 had identical formulations and components, but were prepared on separate dates. b) Reproducibility across separate acquisitions of agar from the same source. In this serial dilution plating assay, we tested a second supply of BD agar that was provided to us by the vendor; we added a letter to the vendor's lot number to distinguish the separately obtained lots of agar (0259186-A for experiments in “a” and 0259186-B for those in “b”).

In a separate, different line of research in a third laboratory, we also encountered—and diagnosed independently—agar lot-specific inhibition of growth on minimal media, this time using the budding yeast *S. cerevisiae*. As with the 2 fission yeast projects presented above, we systematically tested various components of the media for potential toxicities or deficiencies. Strikingly, 4 aspects of the growth inhibition in budding yeast matched those that we documented for fission yeast: First, the inferred toxic agent is traceable unambiguously to specific lots of agar, not to deficiencies in other components of the media (e.g. Fig. 6a, top row). Second, while there was good reproducibility plate-to-plate for a given batch of media, we observed variable penetrance batch-to-batch, even when the formulation and individual components of the media were identical (Fig. 6a, column 3). Interestingly, for the batches of media with low penetrance of toxicity (i.e. high EOP in the presence of toxic agar), we frequently observed a gradient of colony growth across the plate (Fig. 6a, row 2, column 3). This pattern affected equivalently all plates in the stack of poured plates from a given batch of media. These findings are consistent with—and specifically implicate the agar as being causally related to—evidence that exposure of minimal media plates to light while they are solidifying or being incubated can induce toxicity (Alex Nguyen, personal communication; John McCusker, personal communication). Third, the toxicity toward budding yeast, like that for fission yeast, affected multiple, separately acquired lots of agar. For example, when we reported a problem to the vendor, BD, they sent a replacement lot of the agar. That replacement agar also strongly inhibited the EOP of budding yeast, relative to control media whose only difference was the lot of agar (Fig. 6b). The fourth parallel between data for budding yeast and fission yeast, described below, is that the agar lot-specific toxicity appeared in minimal media, not rich media.

In summary, there is strong concordance between discoveries made independently in our 3 different laboratories using 2 distinct, highly diverged species. The data presented here (using 10 distinct lots of agar from 7 different suppliers) are supported further by our data not shown and by unpublished observations shared generously by other researchers. The overall conclusions and their broader implications are striking: The sporadic contamination of agar by toxic agents is relatively common, has occurred repeatedly over multiple decades of agar production, and affects species that are about as distant evolutionarily from each other as humans are from nematodes.

### Rich media attenuates toxicity of the agar

**Fig. 7.**
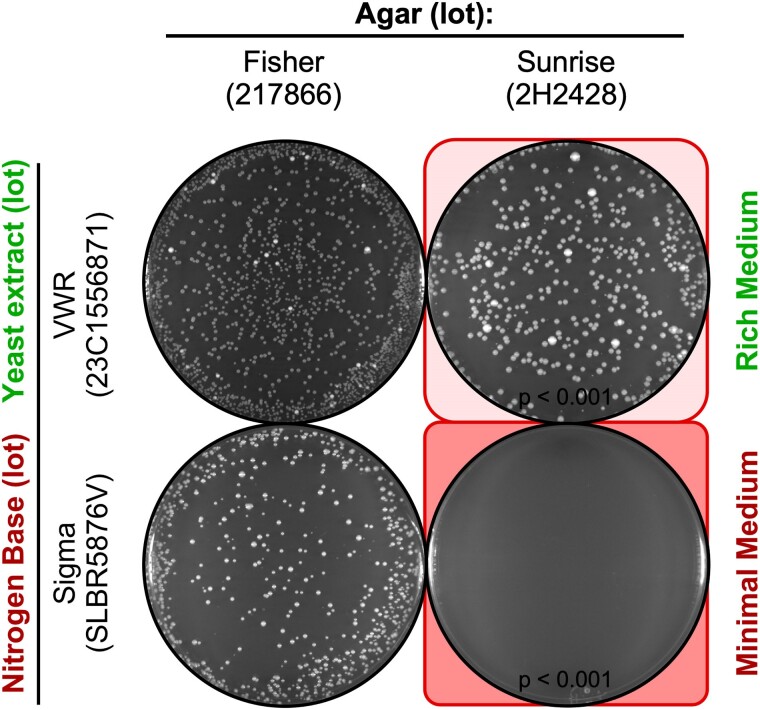
Rich media attenuates the agar lot-specific reduction in plating. Rich YEA (YE) plates and minimal NBA plates were prepared using the indicated lots of agar. In each experiment, 2,500 fission yeast spores (from mating of strains WSP 5189 and WSP 7850) were plated. Representative examples show that the agar lot-specific, reduced spore plating efficiency (bottom right plate) is largely suppressed when the same agar is in media that contains YE (top right plate).

The toxicity of agar toward fission yeast can be suppressed partially by some lots of NB, with variable penetrance (Figs. 2–4), suggesting that some lots of NB have a more abundant counteracting factor (“antidote”) than others. To see if the richness the nutrients might be responsible, we replaced the NB and ammonium sulfate with YE (Fig. 7). This change greatly suppressed the toxicity of the agar, relative to the EOP for controls with nontoxic agar. For example, on toxic agar of lot 2H2428 the EOP was near 0 for media whose nutrient was NB lot SLBR5876V (Fig. 7, bottom right) but the EOP was about 35% for media whose nutrient was YE lot 23C1556871 (Fig. 7, top right). The same principles apply for budding yeast; agar lot-specific inhibition in the EOP affected cells grown on NB minimal media (SD/NB) and was suppressed on rich medium that contained YE (YPD) (our data not shown and Alex Nguyen, personal communication).

The broader implications are striking: Quality control tests that employ only the ability of yeast spores or cells to form colonies when streaked or spread on rich media can give false-positive assurances as to the quality of the agar; correspondingly, such tests can yield false-negative conclusions for the presence of toxicity. Moreover, even on minimal medium, the “Dr. Jekyll vs Mr. Hyde” manifestations of variable penetrance can confound the assays.

## Discussion

### Characteristics, costs, and challenges of toxic agar

This study revealed that some lots of agar contain a yet-unidentified agent that inhibits the growth of fission yeast and budding yeast colonies (Figs. 1–7). The inferred toxic agent suppresses the plating efficiency of both spores and vegetative cells (e.g. Figs. 2 and 4). The toxicity is manifest in multiple different lots of agar from the same vendor (e.g. Figs. 1 and 2; also Fig. 6a vs b), in lots of agar from different vendors (Figs. 4–6), and in agar lots manufactured in each of 3 different decades (e.g. Figs. 4 and 5; Nick Rhind, personal communication). These findings support an overarching conclusion: the sporadic, independently arising toxicity of some agar lots toward *S. pombe* and *S. cerevisiae* is fairly common. Agar lot-specific inhibition of growth also affects other species, such as *C. albicans* (John McCusker, personal communication). This concern is not unique to those who culture yeasts because specific types of agar or components therein can also affect adversely the growth of other microorganisms [e.g. (Tanaka *et al*. 2014; Bosmans *et al*. 2016; Rygaard *et al*. 2017; Costa and Kuramae 2021)].

The sporadic toxicity of agar has substantial costs beyond the value of the agar itself. In our case, the reduction in EOP caused by the toxic agars interrupted our research programs, which rely heavily on quantitative plating of yeast cells and spores. This correspondingly delaying new discoveries and jeopardized future renewal of grant funding. Similar concerns apply for other research and biomedical applications of agar. For example, agar toxicity might contribute to false-positive results for assays on the potency of new antibiotics or to false-negative results when testing the motility of pathogens. Similarly, while the US Food and Drug Administration and other regulatory agencies list agar as being generally recognized as safe, it is possible that a sporadic contaminant that is potent enough to affect adversely the growth of bacterial and yeast cells might also affect mammalian cells.

Our findings revealed 2 main challenges to identifying and tracing toxicity: First, the problem is sporadic and not attributable to a specific vendor (e.g. Figs. 4–6). This is not surprising, given that the production of agar is a worldwide endeavor that has historically relied on many small-scale enterprises (individuals or groups) harvesting many different species from wild sources of seaweed (Porse and Rudolph 2017). Similarly, the manufacture of the agar is decentralized, with many producers and distributors. More fundamentally, the quality of agar is affected by high heterogeneity of numerous factors, including species, growing environments, harvesting and extraction methods, post-extraction treatments, complexity of carbohydrates, their diverse modifications, and contaminants such as fatty acids, phycobiliproteins, pigments, and secondary metabolites [see (Li and Liu 2022) and refs therein]. Toxicity might also stem from chemical contaminants or from other organisms (e.g. bacteria, fungi, and protozoa) on or in the harvested seaweed. And while vendors of scientific agar seek to remove factors that can inhibit microbial growth, the manufacturing processes are proprietary (i.e. opaque), the vendors do not reveal key variables such as species source, and details about quality control measures are typically not provided to end users. Several of these issues could be addressed satisfactorily by simple improvements to quality controls (described below).

The second key challenge identified by this study is that the toxicity displays variable penetrance that can be modulated by the nutrient components of the media (e.g. Figs. 2–4) and can be suppressed substantially by rich nutrients (Fig. 7). This challenge, like the sporadic nature of toxicity (above), complicates the process of identifying the source(s) of problems with culture media: To reveal unambiguously that a problem with the culture media is caused by the lot or batch of agar, one must systematically alter—one variable at a time—both the type of agar and the type of nutrient. Moreover, if a vendor (or an end user) “simply” wished to test whether or not an agar lot is toxic, the choice of nutrient media would be crucial. Scoring only for colony growth on rich media—which suppresses the toxicity of the agar (Fig. 6)—can yield false-negative results for the detection of toxicity. Thus, for quality control tests to be valid, they would have to be conducted using media whose nutrient components permit (i.e. do not mask) the agar toxicity (see recommendations in next section).

### A call to improve quality control measures

Ultimate responsibility for the quality of scientific agar lies with the vendor. A fundamental, broadly applicable finding of this study is that for quality control tests to be valid, they must include quantitative measurements of the EOP on all types of media typically employed for each organism of interest. For *S. pombe*, this would include measuring the EOP of both spores and vegetative cells on rich media (e.g. YEA) and minimal media (e.g. NBA and EMMA). On behalf of scientists worldwide, we call on vendors of scientific agar to conduct—and to document in writing the results of—rigorous, thorough, organism-specific tests for potential toxicity of each lot of agar as a precondition for its sale.

We also encourage members of research communities to be vigilant and proactive. If one encounters newly arising difficulty in plating a given organism, one should suspect—and test for—toxicity within the agar. This could be done expeditiously and inexpensively by comparing growth within liquid media to that on solid media. Confirmed or suspected problems with agar should be brought as soon as possible to the attention of the vendor and members of the relevant research community (e.g. via the PombeList email server). These actions will help colleagues to avoid wasting their precious time and resources and will help the vendors to identify and correct potential defects in their products.

### Concluding perspectives: likely mechanisms and potential benefits of toxicity

Based on our findings and those described to us by others, we can speculate about the nature of the inferred toxin and its antidote. Rich nutrients—an antidote—can repress a variety of transporters, suggesting that activity of the toxin requires its transport into the cell [e.g. (Cummins and Mitchison 1967; Ruiz *et al*. 2020; Patriarcheas *et al*. 2023)]. Thus, the toxin is hypothetically a water-soluble, small molecule or metabolite that preferentially gains access to intracellular target(s) via transporter(s) when they are most active. Reports that exposing petri plates to light can induce a reduction in EOP suggest that the responsible compound might stem from a protoxin that is photoreactive or is processed by some other photoactivatable catalytic process within agar media. Looking forward, the powers of yeast genetics and classical fractionation of bioactive compounds provide complementary approaches to identify the responsible agent(s) and biochemical mechanisms of action. Lastly, we note that the cloud of “bad agar” has a silver lining because if the inferred toxin can be identified and isolated (or manufactured), it could become a therapeutically and agriculturally valuable antifungal agent.

## Data Availability

All data necessary to support the conclusions of this study are contained within the article. Yeast strain reagents are available from the authors upon request.
